# Quaternized Chitosan Thiol Hydrogel-Thickened Nanoemulsion: A Multifunctional Platform for Upgrading the Topical Applications of Virgin Olive Oil

**DOI:** 10.3390/pharmaceutics14071319

**Published:** 2022-06-22

**Authors:** Ali M. Nasr, Salama M. Aboelenin, Mohammad Y. Alfaifi, Ali A. Shati, Serag Eldin I. Elbehairi, Reda F. M. Elshaarawy, Nashwa H. Abd Elwahab

**Affiliations:** 1 Department of Pharmaceutics, Faculty of Pharmacy, Port Said University, Port Said 42526, Egypt; 2 Biology Department, Turabah University College, Taif University, Taif 21995, Saudi Arabia; s.aboelenin@tu.edu.sa; 3 Biology Department, Faculty of Science, King Khalid University, Abha 9004, Saudi Arabia; alfaifi@kku.edu.sa (M.Y.A.); aaalshati@kku.edu.sa (A.A.S.); serag@kku.edu.sa (S.E.I.E.); 4 Cell Culture Lab, Egyptian Organization for Biological Products and Vaccines (VACSERA Holding Company), 51 Wezaret El-Zeraa St., Agouza, Giza 12654, Egypt; 5 Department of Chemistry, Faculty of Science, Suez University, Suez 43533, Egypt; 6 Institut für Anorganische Chemie und Strukturchemie, Heinrich-Heine Universität Düsseldorf, 40225 Düsseldorf, Germany; 7 Department of Pharmaceutics and Industrial Pharmacy, Faculty of Pharmacy, Sinai University—Kantara Branch, Ismailia 41636, Egypt; nashwa.abdelwahab@su.edu.eg

**Keywords:** olive oil, hydrogel-thickened nanoemulsion, quaternized chitosan thiol, antimicrobial and anti-biofilm, antioxidant

## Abstract

(1) Background: Virgin olive oil (VOO) has attracted the attention of many researchers due to its nutritional and medicinal values. However, VOO’s biological applications have been limited due to a lack of precise chemical profiling and approach to increase the physicochemical characteristics, bioactivity, and delivery of its bioactive components; (2) Methods: The current study intended to evaluate the chemical composition of VOO using the GC-MS technique and determine its major components. Furthermore, the effect of incorporating VOO into Tween 80-lecithin nanoemulsion (OONE) and a quaternized trimethyl chitosan-thiol (TMCT) hydrogel-thickened nanoemulsion system (OOHTN) on its physicochemical characteristics and biological potentials will be investigated; (3) Results: The VOO-based NEs’ physicochemical properties (particle size and zeta potential) were steady during storage for four weeks owing to the inclusion of the protective TMCT hydrogel network to OONE. Excessive fine-tuning of olive oil nanoemulsion (OONE) and the TMCT protective network’s persistent positive charge have contributed to the oil’s improved antimicrobial, anti-biofilm, and antioxidant potentials; (4) Conclusions: The Tween 80-lecithin-TMCT nanosystem might provide a unique and multifunctional nanoplatform for efficient topical therapy as well as the transdermal delivery of lipophilic bioactive compounds.

## 1. Introduction

The interest in the encapsulation and delivery of lipophilic biological molecules has led to an increase in the usage of nanoemulsions (NEs) to generate nanoformulations of bioactive lipophilic materials, essential oils, proteins, vaccines, antimicrobials, and so on [1]. Developing essential oil nanoformulations (EONEs) is an alternate strategy that boosts their physical stability, protects them from environmental interactions, and reduces their volatility [2]. Furthermore, essential oil-based nanoemulsions aid in the enhancement of the therapeutic and functionality of the essential oil (EO). For example, when compared to EO, the antimicrobial potential of EONEs was greatly enhanced due to their ability to create a niche over the lipid membrane of microbes via electrostatic attraction, destabilizing the lipid layer through fatty acid solubilization, disrupting protein, and inducing microbial cell death [3]. However, the low viscosity of the nanoemulsion promotes its quick-drying phenomena, resulting in the rapid evaporation of volatile bioactive components after being applied topically. Even though surfactants are commonly used to stabilize NEs, the stability of these nanoformulations may decline over time, leading to coagulation, sedimentation, creaming, flocculation, and the loss of their functional potentials as a consequence [4]. These are thought to be the biggest problems with using essential oil-loaded nanoemulsions as pharmaceutical formulations. 

According to recent research, incorporating nanoemulsion into biopolymeric hydrogels could be a promising approach to addressing these issues [3,5]. In this regard, a novel approach (nanoemulsion-filled hydrogels or hydrogel-thickened nanoemulsions (HTNs)) has recently been proposed as a promising topical delivery system for volatile essential oils due to their good stability, excellent skin permeability, and appropriate viscosity, as well as the potential to improve their performance for topical delivery [3,5,6].

The incredible properties and potentials of chitosan (CS) and its derivatives [7,8], as well as their ability to form extremely stable protective coatings for emulsion systems via electrostatic interactions with anionic surfactants [9], make them appealing platforms for developing stable nanoemulsion delivery systems (NEDSs). To name a few, chitosan plays an important synergistic role in the formulation of *Pelargonium graveolens* essential oil (PGEO) hydrogel-thickened nanoemulsion by enhancing its physical stability (positive zeta potential) and promoting its antimicrobial potential (minimal inhibitory concentration (MIC) values 64 times lower than PGEO) for the treatment of vaginal candidiasis [6]. In another work, chitosan was used as a thickening polymer to formulate clove essential oil (CEO) hydrogel-thickened nanoemulsions (HTNs) for effective transdermal delivery of 8-methoxsalen (8-MOP) [10].

Amongst essential oils (EOs), olive oil (*Olea europaea* L.) has attracted the attention of many researchers due to its nutritional and medicinal values, as well as its organoleptic properties [11]. The consumption of virgin olive oil (VOO) in one’s diet is often associated with lower incidences of atherosclerosis, cardiovascular and neurological disorders as well as certain cancer types [12,13,14]. These beneficial effects are mostly due to the presence of numerous phenolic components, monounsaturated fatty acids (MUFA), and polyunsaturated fatty acids (PSFA), which play an important role in the olive oil’s taste and flavor [15]. Recent studies have demonstrated the effective roles of edible chitosan/olive oil coatings in increasing the shelf life of cold-storage strawberries [16], preserving the quality of stored tomatoes [17], improving fresh fig postharvest quality and prolonging its shelf life [18], and zoonotic skin Leishmaniasis therapy [19]. So far, no report has been published on the formulation and use of chitosan thiomer hydrogel-thickened nanoemulsions incorporating VOO, targeting improving the antimicrobial, anti-biofilm, antioxidant, and transdermal characteristics of olive extract.

Thanks to the amazing structural and biological characteristics of chitosan thiomer (CST) [20], silver nanoparticles (AgNPs) [21], CST-Ag nanocomposites [22], and VOO, coupled with our continuous interest in developing novel safe pharmaceutical formulations [8,23,24], the goal of this study is to evaluate the performance of a new (CST-AgNPs) nanocomposite hydrogel as a thickening agent in formulating a hydrogel-thickened nanoemulsion (HTN) containing VOO. Moreover, the antimicrobial, biofilm inhibition, and antioxidant potential of the novel HTN will be investigated.

## 2. Materials and Methods

The electronic supplementary material (**ESM**†) offered information on the providers of the chemicals that were utilized in this research along with the particulars of the chemicals’ specifications. In addition to this, it detailed the analytic methods that had been implemented in order to completely characterize the materials that had been obtained. The squid pens chitin, ultrasound-assisted deacetylated chitosan (UCS), and its low molecular-weight form (LMWUCS) were obtained from our previous work [25]. Moreover, the preparation of quaternized N-trimethyl chitosan chloride (TMC), from UCS, was depicted in the **ESM†** [22].

### 2.1. VOO Extraction

#### 2.1.1. Sampling

The fruits of Arbequina Olive were cultivated in Egypt in season 2019 in the region of Sheikh Zuid Station, North Sinai Governorate-Center for Desert Research (NS). They were harvested in October–December 2019 and identified by the province in which they were grown (NS). The fruits were stored in dark bottles at 4 °C until they could be used.

#### 2.1.2. Oil Extraction

An Abencor laboratory oil mill equipped with a crusher, mixer, and basket centrifuge was used to extract the oil from the olive fruits. Only fruits that were free of disease or physical harm were used in the processing. After harvesting, the fresh olive fruits were washed with water, dried in air, and then crushed with a hammer crusher operated at 3000 rpm. The paste was thoroughly blended in a mixer at 1400 rpm and 25 °C for 60 min and then subjected to centrifugation using a two-phase centrifugal decanter working at 1372× *g*, to separate the paste into oil and pomace [26]. Finally, the residual suspended solids in the oil were removed using a horizontal centrifuge (4732× *g*) at 40 °C. After filtration of the oil samples through anhydrous Na_2_SO_4_, they were stored in dark glass bottles at −18 °C until they were analyzed or used.

#### 2.1.3. GC-MS Analysis

The chemical composition of the extracted VOO was investigated by the solid-phase microextraction–gas chromatography–mass spectrometry (SPME/GC/MS) technique using a Varian 4000 GC/MS mass spectrometer [27]. A capillary column VF 5ms (60 m × 0.25 mm ID, 0.25 μm film thickness) was used to fractionate the oil samples. Operating conditions were as follows: split ratio, 50:1; He gas flow 1.5 mL/min; injection volume 1.0 μL; column temperature maintained at 40 °C for 10 min, then raised to 180 °C at 20 °C/min, then raised to 220 °C at 10 °C/min; injector, transfer line, and ion source at temperatures were 250 °C, 270 °C, and 200 °C, respectively; pre-incubation time 20 min at 40 °C, and desorption time 5 min. The mean of the data was calculated from three biological repeats obtained from three independent experiments. Electron impact mass spectra (EI-MS) were recorded at ionization energy of 70 eV, 2 scan/s. The VOO’s chemical ingredients were identified by matching the mass spectral patterns and retention times of these ingredients to those of standard compounds or by comparing their mass spectra to those in the Wiley 6th edition mass spectra collection. In addition, the Kováts indices were calculated and compared to published retention indices. Compounds were quantified based on their area in the chromatogram.

### 2.2. Preparation of Trimethyl Chitosan Thiol (TMC-Thiol) Hydrogel (TMCTH)

TMC-thiol was initially prepared using a protocol modified from the method used in our previous study [22], in brief, an aqueous solution (1.5 mL) of thiourea (1.1 g) was acidified with 0.67 mL of 3 M HCl and then added gradually under vigorous stirring to a solution of TMC (1.0 g) in deionized water (DIW) (50 mL). The mixture was then microwaved (640 W) for 5 min with 10 s intervals of irradiation to produce the TMC-isothiouronium intermediate. After that, the intermediate was hydrolyzed by elevating the pH of the reaction mixture to 9.5–10 by the addition of 0.2 N NaOH solution followed by microwave irradiation for 90 s at 640 W. After taking the content out of the microwave oven, it was allowed to cool to room temperature. After neutralizing the cooled solution with 0.1 M HCl, ethanol was added for precipitation of the desired product. The resultant off-white solid (TMC-thiol) was recovered by filtration, washed with acetone (3 × 10 mL), and dried overnight at 50 °C.

The oxidative cross-linking gelation approach was used to covert TMC-thiol to the desired hydrogel (TMCTH). Briefly, TMC-thiol was dissolved into the phosphate saline buffer (PSB) (pH 7.4) to obtain a solution with a concentration of 2% (*w/v*). The reaction mixture was incubated at 37 °C for 1 h to achieve the sol-gel conversion process, as indicated by the observed lowering in the solution fluidity. Because of the ongoing air oxidation process, the solution gradually became more viscous, (i.e., more gel).

### 2.3. Preparation of VOO Primary Nanoemulsion (OONE)

The target NE was prepared using a low-energy self-emulsification process [28], with little modification. Tween 80 was served as a non-ionic surfactant, whereas lecithin (Lec) was used as an ionic co-surfactant, VOO was used as the oil phase, and water was used as the exterior phase. The oil phase (oil-Tween 80) was gradually injected into an aqueous phase (Lec, 10%, *w/v*) while being agitated vigorously (1000 rpm) at ambient temperature, to obtain a final mix of 10% VOO, 40% Tween 80, and 4% Lec in the primary nanoemulsion. The mixture was then stirred for another 15 min under the same conditions. After that, the resultant NE was diluted with an equal volume of DIW and stirred for an additional 30 min to obtain OONE with a final composition of 5% VOO, 20% Tween 80, and 2% Lec.

### 2.4. Preparation of Olive Oil Hydrogel-Thickened Nanoemulsion (OOHTN)

The as-prepared hydrogel (TMCTH) (2%, *w/w*) was gradually added to the nanoemulsion (OONE) while stirring at 500 rpm until obtaining a homogenous dispersion. After that, lactic acid was used to adjust the pH of the reaction mixture. The content was then stirred (500 rpm) at ambient conditions for 30 min to obtain the desired HTN (OOHTN) and kept under refrigerated temperatures for the subsequent experiments.

### 2.5. In Vitro VOO Release

Following a protocol modified from the method described by Muzzalupo et al. [29], the in vitro release of VOO from OONE and OOHTN was studied in phosphate-buffered saline (PBS) (pH 7.4). In brief, 2 mL of the tested sample was put into a glass vial and then diluted with 20 mL of PBS. After that, the diluted sample was agitated at room temperature at a rate of 200 rpm for 48 h. At regular intervals, 2 mL of each sample was taken out and centrifuged at 10,000 rpm. The withdrawn portion was replaced with the same amount of PSB. Next, 1 mL of the resultant supernatant was spectrophotometrically analyzed at 280 nm for quantification of the VOO content. The percent of VOO released was calculated using the equation (Equation (1)):

(1) $$\mathrm{Release}\left(\%\right)=\overset{t}{\underset{t=0}{\sum }}\frac{C_{t}}{C_{0}}\times 100$$

Heir, C_0_, and C_t_ are the initial and released amounts (mg/mL) of VOO at each sampling time, determined from the calibration curve of UV–absorbance against VOO concentrations (Figure S2, **ESM†**).

### 2.6. Ex Vivo Skin Permeability Study

Rat skin (RS) was used as a natural membrane to test the capacity of OOHTN to breach the skin barrier. The Egyptian National Hepatology and Tropical Medicine Research Institute’s Ethics Committee (NHTMRI) has approved the ex vivo skin permeation study procedure. Three- to four-month-old white albino rats that weighed between 250 and 350 g were taken from the animal house at the National Research Center (NRC), Egypt. Rats were sacrificed, and then the abdominal skin was removed, and the hair on the skin was shaved off with an electric clipper. Subcutaneous tissues were then surgically removed once the skin was removed. A saline solution was then used to submerge the skin, with the stratum corneum (SC) facing up. The submerged RS was kept in the freezer until needed. Ex vivo skin permeability experiments were carried out using the Franz diffusion cell (FDC) with an effective diffusion area of 2.44 cm^2^. The excised RS was put between the donor and receiver chambers of the FDC, keeping the SC facing the donor compartment. The receiving chamber was filled with a continuously stirred (500 rpm) PBS solution (pH 7.4) and held at 37 °C. On the other hand, 1 mL of the OOHTN sample was placed in the donor chamber, which was then covered with Parafilm. A 0.5 mL aliquot of the receptor fluid was taken at regular intervals (0.5, 1, 2, 4, 6, 12, 24, and 48 h) and evaluated for VOO concentration to characterize the skin penetration kinetics. Equivalent quantities of fresh PBS were then refilled. Eventually, the cumulative OOHTN permeation (Q_n_, mg/cm^2^) via the skin was determined using the equation (Equation (2)):

(2) $$Q_{n}=\frac{C_{t}V_{r}+\sum_{i=0}^{t-1}C_{i}V_{s}\;}{A}\;\;\;\;\;\;\;\;\;\;\;\;\;\;\;\;$$

C_t_ is the VOO concentration in the receptor compartment at different sampling times, C_i_ is the VOO concentration in the receptor compartment at the i-th (t − 1) sampling time, V_r_ and vs. are the volumes of the receptor compartment solution (12 mL) and withdrawn sample (0.5 mL), respectively, and A is the effective diffusional area [30].

### 2.7. Cytotoxicity Study 

The impact of the new nanoformulations on the viability of the normal human skin fibroblast (HSF) cells was investigated, to validate the potential of using them for safe topical application. The routine MTT method [31] was used for conducting this study.

### 2.8. Antimicrobial Study

According to our previously published antimicrobial test procedure [32], we examined the native oil, OONE, TMCT, and OOHTN for their antimicrobial activity. For this study, *Staphylococcus aureus* (*SA*, RCMB 000106); *Escherichia coli* (*EC*, RCMB 000103); *Aspergillus flavus* (*AF*, RCMB 02782); and *Candida albicans* (*CA*, RCMB 05036) were used as microbial strains to perform this study and were provided by VACSERA, Egypt. Ciprofloxacin HCl (Cipro) was used as a positive control. 

### 2.9. Anti-Biofilm Activity

In accordance with our previously published work [22] with a little change, we performed the anti-biofilm experiment on the novel materials. In brief, a 96-well microtiter plate was filled with aqueous sample solution (10 μg/mL) of the tested sample (30 µL/well) and dried overnight at 37 °C. Deionized water was served as a negative control. TSA (tryptic soy agar) was used as a cultivating medium to grow SA and EA overnight at 37 °C. For each microbial species, a few colonies were dissolved in a TSB enriched with 2% glucose. After being vortexed for 60 s, the optical density at 600 nm (OD_600_ nm) was set to be 0.08, which corresponds to a concentration of about 10^6^ CFU/mL microbial cells in the suspension. Afterward, each pre-coated well received 200 µL of the diluted bacterial solution, whereas wells filled with a non-inoculated TSB medium were served as growth controls. Finally, the plate was kept in an incubator for 24 h at 37 °C. After that, the number of biofilms was determined by following the crystal violet staining process and determining the absorbance of the samples using a microplate reader at 600 nm.

### 2.10. Free Radical Scavenging Activity (DPPH Assay)

The antioxidant activity of free VOO, OONE, TMCT, and OOHTN was evaluated using the 2,2-diphenyl-1-picrylhydrazyl radical scavenging activity (DPPH-RSA) technique reported by Kamal et al. [25] with minor modifications. An aliquot of 0.5 mL of each sample solution of successive concentrations (5–320 µg/mL) was blended with an ethanolic DPPH solution (0.1 mM, 2.5 mL) in test tubes and then the mixtures were agitated and maintained in a dark area for 1 h at 25 °C. DPPH solution was employed as the standard for the experiment. In each experiment, the absorbance was measured at 515 nm and the following equation (Equation (3)) was used to compute the RSA percentage:

(3) $$\mathrm{RSA}\%=\frac{A_{0}-{\;A}_{s}}{A_{0}}\times 100\;\;\;\;\;\;\;\;\;\;\;\;\;$$

where A_0_ and A_s_ are the absorbances of the control and sample, respectively. EC_50_ (μg/mL DPPH) was calculated by interpolation of the linear regression analysis.

### 2.11. Statistical Analysis

Experiments were conducted three times each, and the findings were given as an average ± SD. The OriginPro 9.1E software (version 91E, OriginLab, Northampton, MA, USA) was used to graph the obtained results. For mathematical handling and pairwise comparisons, a one-way analysis of variance (ANOVA) test was used in conjunction with Tukey’s multiple comparison post hoc tests (SPSS software version 22. Chicago, IL, USA). There were only statistically significant differences if the *p*-value was less than 0.05.

## 3. Results and Discussion

### 3.1. Chemical Characterization of Oil

The GC-MS chromatogram of VOO (Figure S1, **ESI†**) has identified 47 major chemical ingredients, which are listed in Table 1. These substances account for 99.29% of the total peak regions on the GC chromatogram. According to Table S1 (**ESI†**), the main volatile constituents of VOO can be categorized as esters, which account for ~24% of oil content, with 3,5-Di-t-butyl-1,4-dihydro-phenacetate, Limonen-6-ol, pivalate, and glafenin as the major components; phenols, which account for ~27% of oil content, with 2,6-Di-tert-butylhydroquinone and carvacrol as the major ones.

Other bioactive ingredients also share in the composition of the VOO, such as alcohols, the majority of which is β-sitosterol and make up around 15% of the oil content; aldehydes, the major component in this family is (E)-2-hexenal and it account for about 3% of the oil content; carboxylic and fatty acids make up about 5% of the oil content, with oleic and 9-octadecenoic acids assignable as the major ones; lactones formulate ~8% of the oil ingredients and the sesquiterpene lactone (Isochiapin B) is the major one; terpenes account for ~6% of the oil content, the majority of which is α-Farnesene; ketones account for ~6% of the oil content, the majority of which is Cholestan-6-one. There are other classes in the VOO, however, with minor contributions to the oil content.

### 3.2. Formation Chemistry

**Scheme 1:**
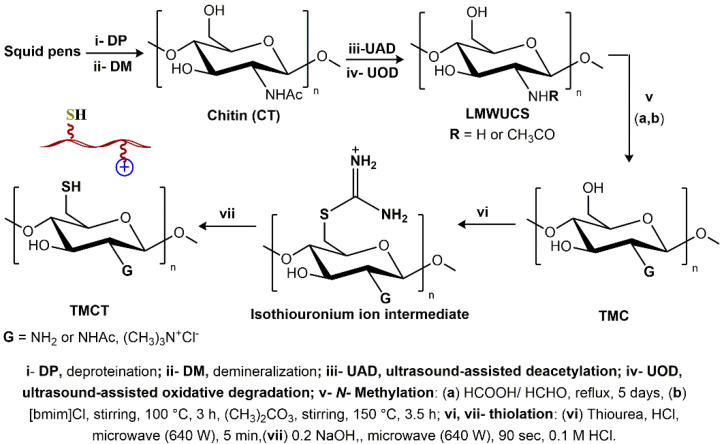
Step-by-step protocol for the preparation of CT, UCS, LMWUCS, TMC, and TMCT.

A set of successive physicochemical processes have been used to fabricate the olive oil-based hydrogel-thickened nanoemulsion (OOHTN). Initially, as depicted in Scheme 1, chitin was extracted from the squid pen wastes using our routine extraction protocol (demineralization (DM) followed by deproteinization (DP)) [33] and then subjected to ultrasound-assisted deacetylation (UDA) process to obtain ultrasound-assisted deacetylated chitosan (UCS). After that, ultrasound-assisted oxidative degradation (UOD) of UCS was performed to obtain low-molecular-weight chitosan (LMWUCS). Afterward, quaternization of the obtained chitosan was performed in two successive steps using an alkylation mixture of formaldehyde-formic acid, followed by (CH_3_)_2_CO_3_ as an N-methylation reagent and in 1-butyl-3-methylimidazolium chloride ([bmim]Cl) ionic liquid as a solvent, to produce quaternized N-trimethyl chitosan chloride (TMC). An indirect thiolation process was used to convert the TMC to TMC-thiol (TMCT). The isothiouronium ion intermediate was first formed by reacting TMC in an acidic medium with thiourea, which then underwent microwave-assisted alkaline hydrolysis to produce the final product. Finally, the oxidative cross-linking gelation process was employed to covert TMC-thiol to the desired hydrogel (TMCTH).

**Figure 1:**
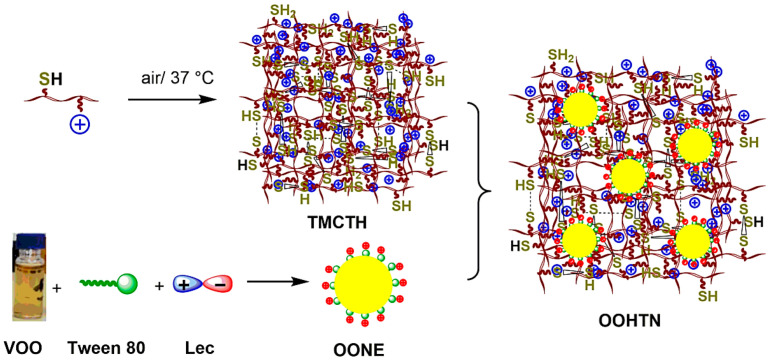
Formulation of OOVE, TMCTH, and OOHTN.

On the other hand, the primary oil nanoemulsion (OONE) was prepared by the physical interactions between the emulsifying agents (Tween 80 as nonionic surfactant and lecithin (Lec) as ionic co-surfactant), oil as an interior phase, and DIW as an exterior phase. Noteworthy, the OONE droplets’ surfaces are negatively charged due to the Lec coating. It was thus possible to form “OOHTN” by the inclusion of the negatively charged droplets of the “primary” nanoemulsion into the positively charged porous network of the TMCTH (see Figure 1).

### 3.3. Physicochemical Characterization

#### 3.3.1. Microanalytical Analyses

The Mark–Houwink–Sakurada (MHS) equation was used to estimate the molecular weights (*M*_w_) of certain Chito-based derivatives from their inherent viscosity ([*η*]) values in aqueous CH_3_COOH/NaCl solutions at 25 °C; [*η*] = *k*·M^α^ where *k* and α are constants (1.81 × 10^−3^ (mL/g) and 0.93, respectively). On the other hand, the degrees of acetylation, quaternization, and substitution (DA, DQ, and DS) has been calculated from the elemental analysis (EA) findings as described in our previous work [22]. Table 1 represents all the microanalytical characteristics of the UCS derivatives.

#### 3.3.2. Infrared Spectroscopy

**Figure 2:**
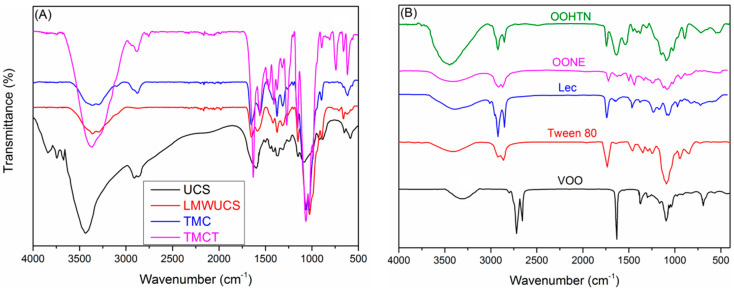
FTIR spectra of (**A**) UCS, LMWUCS, TMC, and TMC-thiol; (**B**) extracted VOO, Tween 80, Lec, primary nanoemulsion (OONE), and its hydrogel-thickened nanoemulsion (OOHTN).

Figure 2A shows the FTIR spectra of the UCS, LMWUCS, TMC, and TMCT, which provide a preliminary indication that our synthesis strategy was successful in producing the intended materials. The stretching vibrations of O–H and N–H, amide I (C=O), N–H bending (NH_2_), and amide II were shown to be responsible for the main LMWUCS characteristic peaks at 3459, 1651, and 1589 cm^−1^. In contrast, the removal of the N–H absorption band at 1589 cm^−1^ in TMC’s spectra supports the substitution of H-atoms in the primary NH_2_ group with CH_3_ groups and N-quaternization of LMWUCS. The appearance of additional absorption peaks at 2683 and 639 cm^−1^ in the TMCT spectrum, which corresponds to the S–H and C–S stretching vibrations of the thiol group, shows that TMCT has been successfully thiolated to create TMCT. The presence of the main characteristic IR peaks of VOO (3438 cm^−1^, alcoholic and phenolic OH; 1744, C=O ester; 1618 cm1, C=C–C; 1331 cm1, phenolic OH), Tween 80 (1744 cm^−1^, C=O ester; 3438 cm^−1^, O–H alcoholic; 3031 cm^−1^, C–H olefinic; 2928 cm^−1^, C–H methyleneic; and 2858 cm^−1^ methyl groups [34], and Lec (1744 cm^−1^, C=O ester; 1239 cm^−1^, P=O; 1547 cm^−1^, (CH_3_)_3_N^+^−; 1069 cm^−1^, P–O–C) [35] in the OONE (VOO-Tween80-Lec) spectrum (Figure 2B), is indicative of its successful formulation. The remarkable alterations in the intensities and/or positions of these IR peaks could be ascribed to the mutual interactions between the components of this nanoemulsion. In a similar scenario, the success of the incorporation of OONE into the matrix of TMCTH was evident by the emergence of new vibration bands which are distinctive of TMCT in the OOHTN spectrum (Figure 2B), in addition to the OONE peaks.

#### 3.3.3. Droplet Size, Polydispersity Index, Zeta Potential, and Storage Stability

**Figure 3:**
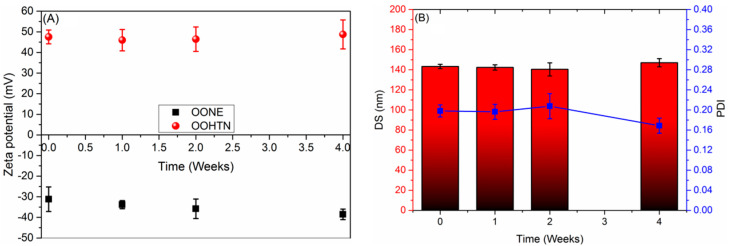

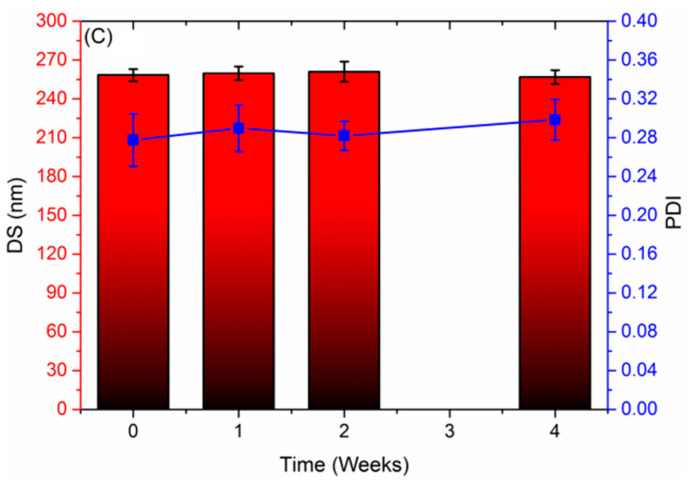
The effect of hydrogel protective network on the storage stability of the OO-based nanoformulations: (**A**) changes in zeta potential (mV) during four-week storage at room temperature; (**B**) changes in droplet size and PDI of OONE with storage time; (**C**) changes in droplet size and PDI of OOHTN with storage time.

The storage stability of the novel nanoformulations was validated by tracking the changes in droplet size (DS), polydispersity index (PDI), and zeta potential (ZP) for these nanoformulations over a four-week period. Figure 3A shows that the zeta potential of primary VOO nanoemulsion (OONE) switched from −31.17 mV to −38.58 mV, while the nanoemulsion droplet size grew from 142.84 to 147.96 nm and the PDI also went up (from 0.16 to 0.23) ((Figure 3B)). An Ostwald ripening of droplets may be responsible for the increase in droplet size [36]. In contrast, OOHTN is more stable than OONE, as evident by the very slight alterations that occurred in the values of droplet size (changed from 258.41 to 257.52 nm) (Figure 3C), PDI (changed from 0.28 to 0.29 nm) (Figure 3B), and ZP (from +47.55 nm to +48.73 nm) (Figure 3C) during storage. In addition, the PDI values remained within the desired range (<0.3) for uniform monodisperse systems [37]. Because the Lec outer layer is negatively charged, OONE has a negative ZP value. In contrast, the positive ZP value for OOHTN is due to OONE’s cationic outer layer being formed by TMCT.

### 3.4. Morphological Characterization

#### SEM Analysis

**Figure 4:**
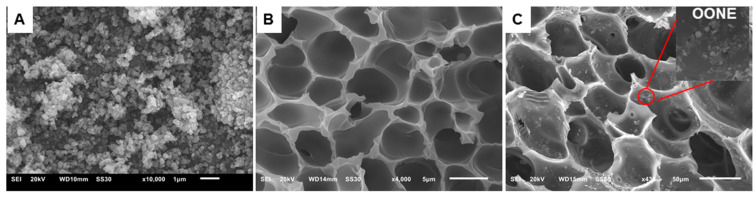
SEM micrographs of (**A**) OONE, (**B**) TMCTH, and (**C**) OOHTN.

The morphological features of the OONE, TMCTH, and OOHTN were inspected using scanning electron microscopy (SEM) (see Figure 4). In OONE, spherical droplets of almost the same size and shape have been formed, as shown in micrograph Figure 4A. The spherical shape of the OONE droplets could be due to their high surface energy, which slowed down the rate of undesirable processes such as creaming and flocculation that often happen in nanoemulsions [38].

The SEM micrograph of hydrogel (TMCTH) (Figure 4B) shows that its inner structure had a highly porous and interconnected pore structure as a result of a various range of crosslinking degrees. This porous structure would be beneficial for the effective entrapment of olive oil nanoemulsion droplets. Even after the addition of OONE, the hydrogel reserves its highly porous structure with tiny droplets entrapped on the inner surface of hydrogel pores, as shown in micrograph Figure 4C.

### 3.5. Pharmacological Performances

#### 3.5.1. Entrapment Efficiency (EE) and Oil Loading (OL)

The estimated EE and OL for OOHTN were found to be 96.1% and 3.7%, respectively, demonstrating that the Tween 80–Lec–TMCTH system will be promising for entrapment and encapsulation of essential oils. This might be ascribed to lecithin’s superior emulsifying characteristics as well as the existence of a persistent cationic charge on the TMCTH surface, allowing it to develop a flexible and effective interaction with the negatively charged Lec surface. As a result, the Lec–TMCTH complex has superior interfacial characteristics than that of its original components. Furthermore, it is possible that the little oil loss (3.9%) is related to the evaporation of oil’s volatile ingredients during the nanoemulsion manufacturing process.

#### 3.5.2. Ex Vivo Skin Permeability Study and Release Kinetics

**Figure 5:**
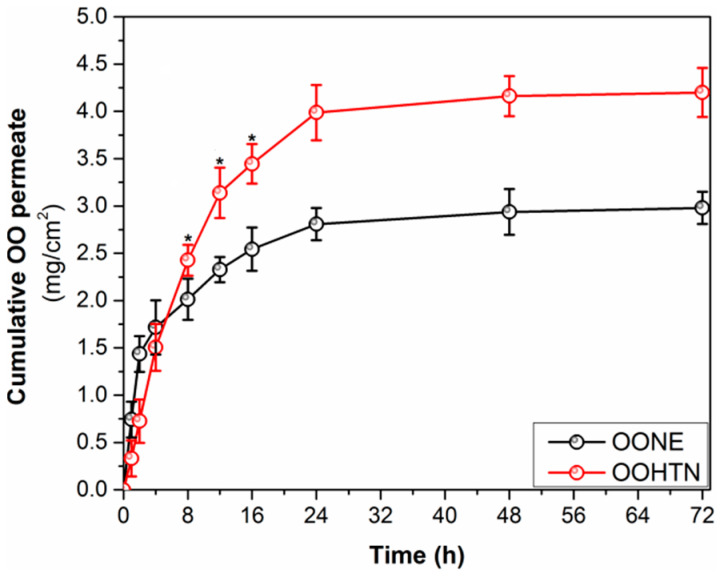
Skin permeability style of OO-loaded nanoemulsions (OONE and OOHTN) through RS. The asterisks (*) represent a significant difference (*p* < 0.05) of OOHTN from OONE.

Because virgin olive oil has a low skin permeability [39], its incorporation into nanoemulsions might be a potential technique for overcoming the problems associated with VOO’s low skin permeability for topical applications. Consequently, the ex vivo capacity of the OOHTN to cross the skin barrier was examined in comparison with the OONE, with the Franz diffusion cell employing rat skin as a naturally occurring barrier. Figure 5 depicts the relationship between the total amount of VOO-based NEs that infiltrates through the unit area of RS and experiment time. The primary nanoemulsion (OONE) exhibited significantly (*p* < 0.05) greater skin permeability (1.43 mg/cm^2^) than the hydrogel-thickened NE (OOHTN) (0.72 mg/cm^2^) in the first two hours. This could be because OONE droplets (143 nm) are smaller than OOHTN ones (258 nm), which restricts the passage of VOO bioactive components through the skin. The permeation style of OOHTN, on the other hand, was superior to that of OONE after 4 h and reached a plateau after 24 h. OOHTN’s skin permeation was 4.15 mg/cm^2^ at the end of the first day, which is considerably (*p* < 0.05) greater than OONE’s 2.71 mg/cm^2^. Consequently, the positive charges on OONTN’s surface were shown to be crucial in increasing its skin permeability by ~1.7 times more than the negatively charged OONE. The increased OOHTN’s skin permeability might be ascribed to the high positive charge density of its nanodroplets (ZP = +47.51 mV), which allows it to adhere strongly to the negatively charged cell membrane of RS, and consequently, provide enough time for the nanodroplets to penetrate the skin. On the other hand, the increased OONE’s negative charge (ZP = −31.17 mV), induces repelling from the negatively charged RS cell membrane [40].

Equations (Equations (4)–(6)) [40] were used to calculate the permeability indices such as steady-state transdermal flux (*J*_ss_, mg/cm^2^h), permeability coefficient (*K*_p_, cm/h), and enhancement ratio (*E*_r_) and the obtained values are presented in Table 2.

(4) $$J_{\mathrm{ss}}=\frac{\mathrm{Slope}\;\mathrm{of}\;\mathrm{the}\;\mathrm{linear}\;\mathrm{part}\;\mathrm{of}\;\mathrm{the}\;\mathrm{graph}\;}{\mathrm{Diffusion}\;\mathrm{cell}\;\mathrm{area}}\;\;\;\;\;\;\;\;\;\;\;\;\;\;\;\;$$

(5) $$K_{p}=\frac{J_{\mathrm{ss}}\;}{\mathrm{Initial}\;\mathrm{concentration}\;\mathrm{of}\;\mathrm{NE}}\;\;\;\;\;\;\;\;\;\;\;\;\;\;\;$$

(6) $$E_{r}=\frac{J_{\mathrm{ss}}\;\mathrm{for}\;\mathrm{NE}\;}{J_{\mathrm{ss}}\;\mathrm{for}\;\mathrm{control}\;\left(\mathrm{TEO}\right)}\;\;\;\;\;\;\;\;\;\;\;\;\;\;\;\;$$

Table 2 shows that the *J*_ss_, *K*_p_, and *E*_r_ values for OOHTN were significantly increased (*p* < 0.05) as compared to the native OO and OONE. For example, the values of permeability indices for OOHTN were (*J*_ss_, 0.2683 mg/cm^2^ h; *K*_p_, 5.41 × 10^−3^ cm/h; *E*_r_, 5.55), which are greatly higher than that of OONE (*J*_ss_, 0.0824 mg/cm^2^ h; *K*_p_, 0.98 × 10^−3^ cm/h; *E*_r_, 1.68).

The OO-based NEs’ release properties were examined by fitting data on skin permeability to several kinetic model equations. The optimum kinetic model was found using the coefficient of correlation (*R*^2^) value. According to the findings in Table 2, the Higuchi model was assigned to be the most suited for describing the time course of olive oil release from its nanoformulations and demonstrating their permeability through diffusion.

#### 3.5.3. Cytotoxicity

Nanoformulations’ effect on human skin fibroblast (HSF) vitality was examined in order to verify their prospective use for safe topically applied treatment. All tested formulations exhibited low antiproliferative effects on the HSF cells as indicated by their increased IC_50_ values, which were quantified as 110.53 ± 3.54 μg/mL, for native VOO; 92.12 ± 3.13 μg/mL, for OONE; and 86.31 ± 2.79 μg/mL, for OOHTN. Thus, OOHTN could offer a promising pharmacological candidate or drug delivery tool for topical therapy. The lower cytotoxicity of new materials could be explained based on the Food and Drug Administration (FDA) regulations which categorized chitosan and olive oil as safe bioactive ingredients [41,42]. In food, the FDA has approved the use of olive oil as an antibacterial agent. Because of its capacity to drive fibroblast proliferation and its significant anti-inflammatory action on the NF-kappa B system, it is also beneficial for tissue repair and wound healing [43]. As a result, we employed the IC_50_, which is harmless on the fibroblast, to apply as an antioxidant, antimicrobial, and anti-biofilm medication. Chitosan and its derivatives, on the other hand, have the ability to stabilize RBC membranes, making them hemostatic [44].

#### 3.5.4. In Vitro Antimicrobial Study

**Figure 6:**
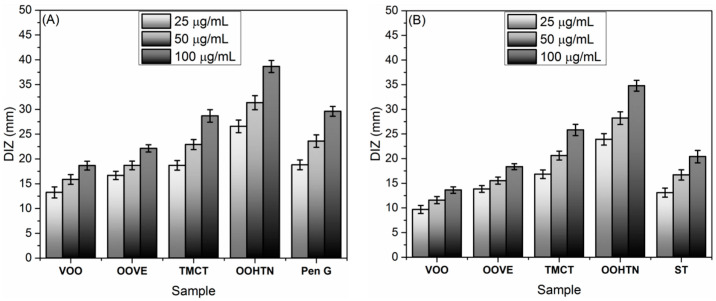
DIZ graph for VOO-nanoformulations and their native ingredients against (**A**) *S. aureus* (*SA*) and (**B**) *E. coli* (*EC*). Pen G = Penicillin G and ST = Streptomycin were used as negative controls.

The antimicrobial properties of VOO and its nanoformulations were evaluated by an agar well-diffusion (AWD) assay using serial concentrations (25–150 μg/mL) of these materials. The broth dilution (BD) technique was used to estimate the minimum inhibitory concentration (MIC) of the microbes. The BD test was performed in a 96-well microtiter plate with samples ranging in concentration from 0.05 to 32 μg/mL. Based on the diameter of inhibition zone (DIZ) values, the new materials had significant inhibitory effects on the tested bacterial species (SA and EC). However, the performance depends on the molecular structure of the sample as well as the type of bacterium. Generally, in comparison to the native VOO and TMCT components, the antibacterial activity of oil following incorporation into nanoemulsions has been dramatically improved. In this scenario, the DIZ values of the OOHTN were in the range of 23.34–38.64 mm which is significantly higher than the DIZ values of its primary ingredients VOO (9.65–18.66 mm), OONE (13.23–22.13 mm), and TMCT (16.68–21.73 mm) (see Figure 6). Meanwhile, the MIC values of OOHTN were in the range of 0.50–1.75 μg/mL which is greatly higher than the MIC values of its starting materials: VOO (23.75–48.75 μg/mL), OONE (14.31–42.48 mm), and TMCT (13.68–28.68 mm). The greater activity of OOHTN compared to its native constituents could be attributed to increased infiltration of the HTN bioactive components (oil biomolecules and biomacromolecules (TMCTH)) and their ability to elicit various bactericidal effects into the bacterial cell. Furthermore, the structural differences between Gram-negative and Gram-positive bacteria’s walls may explain why *SA* is more susceptible to this preferred action. Chitosan-thiol-coated oil is also bioadhesive, making it easier for oil nanodroplets to penetrate the mucus layer of bacteria, allowing them to be more easily absorbed. Noteworthy, most of the samples tested were more effective against *SA* than against *EC*.

As for the antifungal activity evaluation for these materials, VOO-based nanoformulations, in general, have shown a considerable improvement in antifungal efficacy when compared to individual components of each nanoformulation. The DIZ values of VOO, TMCT, and OOTHN were in the range of 19.42–25.35 mm, while the MIC values varied from 7.25 μg/mL to 50.50 μg/mL, against tested fungal species.

#### 3.5.5. Anti-Biofilm Assessment

**Figure 7:**
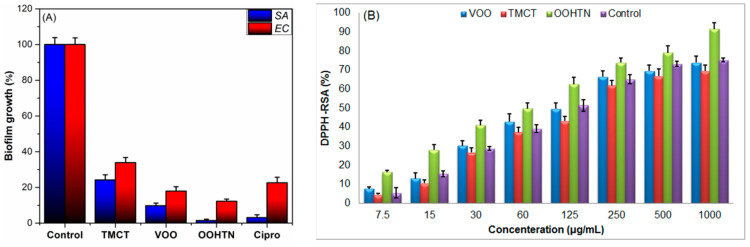
(**A**) Anti-biofilm activity graph for VOO-nanoformulations and their native ingredients against *SA* and *EC*, as compared to Cipro and DIW as a positive and negative control, respectively. (**B**) DPPH-RSA (%) for the same samples, ascorbic acid was used as a control.

In vitro evaluations of the ability of novel HTN (OOHTN) and its native constituents (VOO, TMCT) to restrict the development of *EA* and *SA* biofilms on polystyrene surfaces were compared to biofilm growth in the case of negative control (DIW) and positive control (Cipro). Figure 7A shows that all examined materials had the ability to significantly inhibit the production of bacterial biofilms. For example, all samples may inhibit the production of staphylococcal biofilms more efficiently than *EC* biofilms. One of the most notable findings was that OOHTN had the greatest anti-staphylococcal biofilm activity (only 1.45% ± 0.15 biofilm developments; 98.55% biofilm growth inhibition (BGI)), which was more than the positive control (Cipro) (3.1% ± 1.6% biofilm growth). This improved anti-biofilm activity might be ascribed to the nanoemulsion’s ability to block *SA* cell adhesion to polystyrene surfaces coated with it, along with its extremely effective antibacterial action on *SA* cells embedded in cultures or biofilms. The tested materials inhibited EC biofilm formation also, although with less efficacy when compared to SA. In a similar vein, the order capability in suppressing the formation of EC biofilm is OOHTN (87.89% BGI) > OONE (82.11%) > positive control (Cipro) (77.4%) > TMCT (66.13% BGI) (Figure 7A). The potential mechanism for the higher antibiofilm activities of VOO-based nanoemulsions could be related to their superior ability to inhibit bacterial cell adhesion to polystyrene surfaces coated with these NEs, combined with their extremely potent bactericidal impacts on bacterial cells submerged in cultures or biofilms [22].

#### 3.5.6. Antioxidant Study

VOO, TMCT, and their hydrogel-thickened nanoemulsion form (OOHTN) were tested for their antioxidant activity using the DPPH assay, and the findings are shown in Figure 7B. The DPPH radicals scavenging activity (DPPH-RSA) of the olive oil was significantly increased (*p* < 0.001) after inclusion into the biopolymeric hydrogel network, as evidenced by the findings, which ranged from 7.28–73.95% and 16.74–91.01% for the VOO and OOHTN, respectively. Furthermore, antioxidant activity increased concentration-dependently, and the sequence of antioxidant activity was OOHTN > VOO > TMCT. The IC_50_ values for these materials, which were 223.48, 136.11, and 69.34 µg/mL for TMCT, VOO, and OOHTN, respectively, are further proof of this sequence. The amazing antioxidant activity of Olive oil-TMCT hydrogel could be attributed to the synergistic antioxidant effects of many natural antioxidants in oil [45] and the protective film of chitosan derivative (TMCT) [46].

## 4. Conclusions

Virgin olive oil (VOO) was successfully extracted from fruits of Arbequina Olive and chemically characterized using GC-MS. VOO was then incorporated into a Tween 80 Lec nanoemulsion to formulate the primary nanoemulsion (OONE). On the other hand, quaternized N-trimethyl chitosan chloride (TMC) and its thiolated form (TMCT) were produced from squid pen wastes. TMCT was used to construct a protective hydrogel with a highly porous network to encapsulate OONE droplets, targeting the preparation of olive oil-based hydrogel-thickened nanoemulsion (OOHTN) for potential topical pharmacological applications. The new nanoformulation (OOHTN) was structurally and morphologically characterized. After adding OONE to a protective hydrogel (TMCTH), the droplet size (DS) of NE grew, and the zeta potential changed from negative to positive. Storage stability experiments have shown that the hydrogel has substantially improved the stability of the as-fabricated HTN (OOHTN), allowing it to be kept for up to four weeks without noticeable changes in its characteristics (particle size, PDI, and zeta potential). In addition, the TMCTH has greatly improved the OOHTN’s transdermal delivery. As a result, OOHTN had 1.7 times the transdermal delivery of primary nanoemulsion (OONE). According to the results of the in vitro kinetic release experiment, OO was released from the novel nanoemulsions using the Higuchi model. OOHTN exhibited very low toxic effects on the normal human (HSF) cells. The antimicrobial, anti-biofilm, and antioxidant properties of VOO were dramatically increased after incorporation into the matrix of the highly porous hydrogel (TMCTH).

## Figures and Tables

**Table 1:** Microanalytical properties of UCS derivatives.

Sample	[*η*] (mL/g)	M_w_ (kDa)	EA (%)				DA	DQ	DS
			C	H	N	S			
UCS	488.67	691.6	40.14	7.21	7.65	–	6.2	–	–
LMWUCS	21.18	23.8	40.11	7.28	7.49	–	7.5	–	–
TMC	35.51	41.7	48.87	7.97	6.07	–	–	13.4	–
TMCT	51.85	49.7	42.32	7.49	5.06	23.9	–	–	1.66

**Table 2:** Permeability indices (*J*_ss_, *K*_p_, and *E*_r_) and correlation coefficients for the in vitro release kinetic parameters of olive oil from its nanoformulations.

Sample	Permeate in 24 h (mg/cm^2^ h)	*J*ss (mg/cm^2^ h)	*K*_p_ × 10^−3^ (cm/h)	*E*_r_	*R* ^2^		
					Zero Order	1st Order	Higuchi
**VOO**	–	0.0511 ± 0.02	0.18 ± 0.04	–	–	–	–
**OONE**	2.71 ± 0.11	0.0824 ± 0.04	0.98 ± 0.13	1.68 ± 0.26	0.85374	0.68574	0.96313
**OOHTN**	4.15 ± 0.16	0.2683 ± 0.11	4.41 ± 0.85	5.55 ± 0.76	0.97867	0.80386	0.99982

## Data Availability

Not applicable.

## Supplementary Materials

The following supporting information can be downloaded at: https://www.mdpi.com/article/10.3390/pharmaceutics14071319/s1, Figure S1: GC-MS chromatogram of the extracted VOO, Figure S2: Calibration curve of olive oil (OO)-based nanoemulsion, Table S1: Chemical composition of VOO.

## Abbreviations

VOO, Virgin olive oil; CS, chitosan; CST, chitosan thiomer; EONEs, essential oil nanoformulations; EE, Encapsulation efficiency; EOs, essential oils; HTNs, hydrogel-thickened nanoemulsions; Lec, lecithin; LMWUCS, low molecular-weight ultrasound-assisted deacetylated chitosan; MIC, minimal inhibitory concentration; MUFA, monounsaturated fatty acid; NEs, nanoemulsions; NEDSs, nanoemulsion delivery systems; OC-NE, oligochitosan-coated nanoemulsion; OONE, olive oil nanoemulsion; OOHTN, olive oil hydrogel-thickened nanoemulsion; PSFA, polyunsaturated fatty acids; RS, rat skin; SC, stratum corneum; TMCT, trimethyl chitosan-thiol; UCS, ultrasound-assisted deacetylated chitosan, VOO, Virgin olive oil
