# Supplementary material for: Quaternized Chitosan Thiol Hydrogel-Thickened Nanoemulsion: A Multifunctional Platform for Upgrading the Topical Applications of Virgin Olive Oil
Source: Pharmaceutics. 2022 Jun 22;14(7):1319. doi: 10.3390/pharmaceutics14071319 (PMC9320276; doi:10.3390/pharmaceutics14071319)
Supplement: Supplementary file 1 [file pharmaceutics-14-01319-s001.zip › pharmaceutics-1767266-supplementary.pdf]

Quaternized chitosan thiol hydrogel-thickened nanoemulsion: A multifunctional platform for upgrading the topical applications of virgin olive oil

Ali M. Nasr [1], Salama M. Aboelenin [2], Mohammad Y. Alfaifi[3], Ali A Shati[3], Serag Eldin I. Elbehairi[3,4], Reda F. M. Elshaarawy[5,6,*], Nashwa H. Abd Elwahab[7]

[1] Department of Pharmaceutics, Faculty of Pharmacy, Port Said University, 42526 Port Said, Egypt; a.nasr@pharm.psu.edu.eg.
[2] Biology Department, Turabah University College, Taif University, 21995, Saudi Arabia; s.aboelenin@tu.edu.sa
[3] Biology Department, Faculty of Science, King Khalid University, 9004 Abha, Saudi Arabia; alfaifi@kku.edu.sa; aaalshati@kku.edu.sa
[4] Cell Culture Lab, Egyptian Organization for Biological Products and Vaccines (VACSERA Holding Company), 51 Wezaret El-Zeraa St., Agouza, Giza, Egypt; serag@kku.edu.sa
[5] Department of Chemistry, Faculty of Science, Suez University, 43533 Suez, Egypt; reda.elshaarawy@suezuniv.edu.eg
[6] Institut für Anorganische Chemie und Strukturchemie, Heinrich-Heine Universität Düsseldorf, Düsseldorf, Germany; reel@hhu.de
[7] Department of Pharmaceutics and Industrial Pharmacy, Faculty of Pharmacy, Sinai University – Kantara Branch, Ismailia 41636, Egypt; nashwa.abdelwahab@su.edu.eg

* Correspondence: AMN, a.nasr@pharm.psu.edu.eg; Tel. +2011100089084: RFME, reda.elshaarawy@suezuniv.edu.eg; Tel. +201017377216

.

Contents:

1. **Materials and Instrumentation**
2. **Extraction of chitosan (UCS) and preparation of low molecular weight chitosan (LMWUCS)**
3. **Preparation of *N*-methylated water soluble chitosan (TMC)**
4. **Figures**

1. Materials and Instrumentation

1.1 Materials

Chemicals were obtained from the following suppliers and used without further purification: Grlacial acetic acid (CH_3COOH), Sodium hydroxide (NaOH), Thiourea, and Hydrogen peroxide (30%) (Adwic); Dimethyl carbonate ($(CH_3)_2CO_3$) (99%) 1-butyl-3-methylimidazolium chloride ([bmim]Cl) (96%) (Alfa Aesar); sodium sulphate anhydrous (Na_2SO_4), sodium hydroxide (NaOH) and glacial acetic acid (ADWIC)

1.2 Instrumentation

Elemental analyses for C, H, N and S were performed with a Perkin–Elmer 263 elemental analyzer. FT-IR spectra were recorded on a BRUKER Tensor-37 FT-IR spectrophotometer in the range 400–4000 cm^{-1} as KBr discs or in the 4000-550 cm^{-1} region with 2 cm^{-1} resolution with an ATR (attenuated total reflection) unit (Platinum ATR-QL, Diamond). For signal intensities the following abbreviations were used: br (broad), sh (sharp), w (weak), m (medium), s (strong), vs (very strong). NMR-spectra were obtained with a Bruker Avance DRX200 (200 MHz for ^1H) or Bruker Avance DRX500 (500 MHz for ^{13}C) spectrometer with calibration to the residual proton solvent signal in D_2O (^1H NMR: 4.79 ppm) against TMS with $\delta = 0.00$ ppm. Multiplicities of the signals were specified s (singlet), d (doublet), t (triplet), q (quartet) or m (multiplet). UV-Vis spectroscopy was used to examine the wavelength in nm. The particle shape of new materialswas examined using transmission electron microscope (TEM). The images were taken by a JEM-2011F microscope (JEOL, Japan) operated at 200 kV. The morphology of the formed micro and nano-composites was investigated using Scanning electron microscopy (SEM, Hitachi S-7400, Hitachi, Japan) supported with energy dispersive –X-ray (EDX) to determine the elemental analysis of the formed products.

2. Extraction of chitosan (CS) and preparation of low molecular weight chitosan (LMWUCS)

The squid β-chitin was ground in a ball mill and fractionated using sieves. The fraction with particles of average diameter in the range of 0.125-0.250 mm was subjected to the ultrasound-assisted deacetylation (USAD) process. The β -chitin powder was suspended in an aqueous NaOH solution (10:1, volume (mL)/ mass (g)) and the suspension was conveyed to a double-walled cylindrical glass reactor of internal diameter 3.5 cm and equipped with a circulating thermostat to control the reaction temperature. Then the chitin suspension was subjected to ultrasonic irradiation in a LUHS-A17 sonicator (ν = 20 kHz). Based on the previous studies, the following operating conditions were selected for carrying out an USAD process; (i) β-chitin/NaOH = 1/10 g mL^{-1}, (ii) Suspension volume = 50 mL (iii) Irradiation pulse (IP = 0.5 s), (iv) Irradiation surface intensity (I = 52.6 Wcm^{-2}), (v) reaction temperature (60 ± 1 °C), (vi) Reaction time = 50 min. After completion of reaction duration, the reaction was quickly quenched by cooling the suspension to - 5 °C and neutralized by the addition of dilute HCl to precipitate the USAD product (UCS_1) which was collected by filtration and thoroughly washed with deionized water and freeze-dried. Applying the USAD process on USC_1 for further two consecutive times

resulted in obtaining the disered UCS. After that, UCS was subjected to the oxidative degradation mediated by H_2O_2 and ultra-sonication to form LMWUCS was carried out according to our previously reported protocol [25].

3. **Preparation of N-methylated water soluble chitosan (MWSC)**

3.1. Preparation of N,N-dimethyl chitosan (DMC):

DMC was prepared prior to the synthesis of trimethyl chitosan (TMC). The preparation method for N, N-dimethyl low molecular weight chitosan was first reported by Muzzarelli and Tanfani and subsequently improved by Verheul et al. [22]. Five grams of LMWUCS was dissolved in 15 mL of formic acid, then 20 mL of formaldehyde solution and 90 mL of deionized (DI) water were added to the flask. The solution was heated to 70 °C and was subjected to magnetic stirring and reflux condensation for 5 days. After rotary evaporation at 60 °C for 50 min, the solution was adjusted to pH 13 using a 1N NaOH solution. A large amount of gel was immediately formed, and the solution was adjusted to pH 4 using diluted HCl. The product was filtered using G3 funnels and then freeze dried.

3.2. Preparation of N-methylated (N,N,N- trimethyl) water-soluble chitosan (TMC):

N,N,N-trimehtyl derivative of LMWC (MWSC) was obtained by quaternization of chitosan using dimethyl carbonate ($(CH_3)_2CO_3$) as *N*-methylation reagent in 1-butyl-3-methylimidazolium chloride ([bmim]Cl) ionic liquid [22]. Half gram of DMC was slowly added to 10 g of [bmim]Cl in a three-necked flask (TNF) equipped with a reflux condenser and thermometer. The solution was then heated to 100 °C while magnetically stirred. A homogenous solution formed after 3 hours. Then, 10 g of dimethyl carbonate was added to the TNF and the reaction proceeded at 150 °C under stirring for 3.5 h. The resultant dark brown solution was washed five times with anhydrous ethanol, and then TMC was obtained by drying at 70 °C.

2. Figures Captions

Figure S1: GC-MS chromatogram of the extracted VOO.

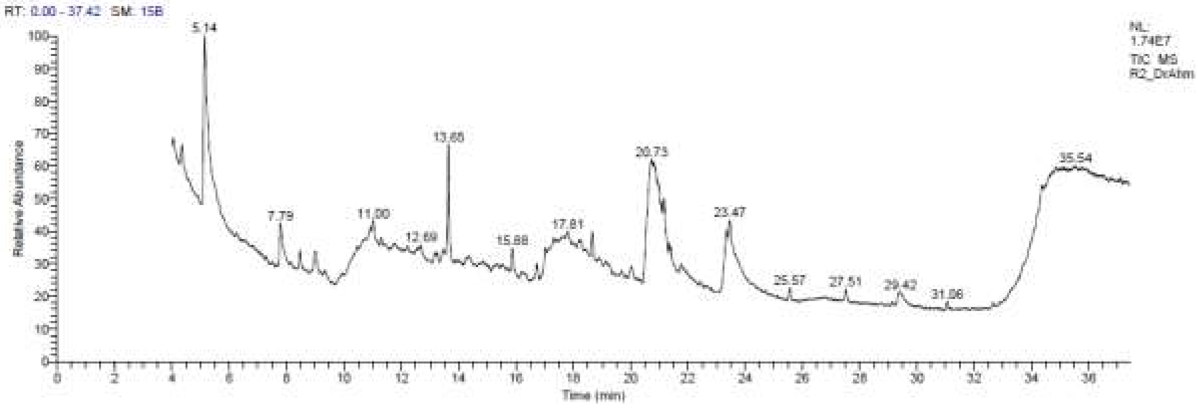

Figure S2: Calibration curve of olive oil (OO)-based nanoemulsion

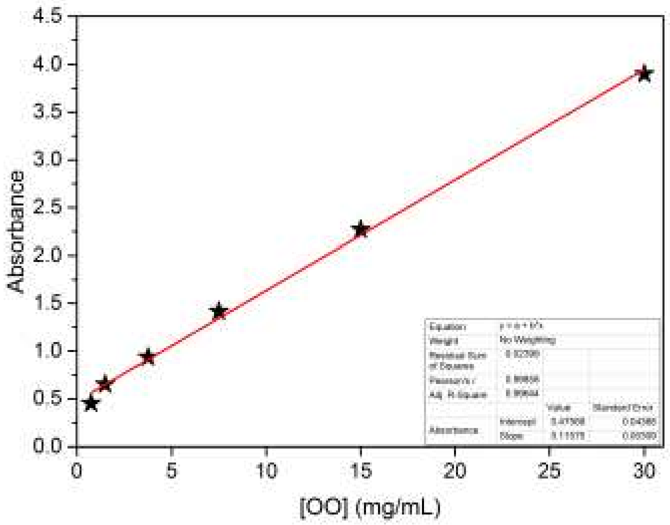

Table S1: Chemical composition of VOO

No.	Compound	RT (min)	Yield (%)	No.	Compound	RT (min)	Yield (%)
**1**	Limonen-6-ol, pivalate	4.05	2.35	**23**	Heptanal	18.28	1.16
**2**	2,6-Di-tert-butylhydroquinone	4.34	2.41	**24**	Z-5-Methyl-6-heneicosen-11-one	18.68	2.93
**3**	2,6-Di-tert-butylhydroquinone	5.12	15.73	**25**	Methyl acetyloxysarpagan-16-carboxylate	19.68	0.99
**4**	Isoamyl isovalerate	6.27	1.16	**26**	(E)-2-hexen-1-ol	20.03	2.05
**5**	Glafenin	7.78	3.93	**27**	Isochiapin B (Sesquiterpen lactone)	20.67	5.81
**6**	Geranyl acetate	8.46	1.82	**28**	Guaiacol	20.82	1.18
**7**	Carvacrol	8.98	3.77	**29**	Isochiapin B	20.87	0.89
**8**	(E)-2-hexenal	9.36	1.30	**30**	Digitoxin	20.99	1.23
**9**	Thymol	10.90	1.15	**31**	1-Heptatriacotanol	21.08	0.80
**10**	p-Cymen-7-ol	11.02	1.67	**32**	Tridecanol	21.17	2.63
**11**	4-Ethyl phenol	11.29	0.83	**33**	Linoleic acid ethyl ester	21.25	0.41
**12**	4-Allylphenol	12.21	1.37	**34**	2-Monoolein (fatty alcohols)	21.37	1.23
**13**	1,2,3-Propanetriol, diacetate	12.68	0.98	**35**	Ethyl iso-allocholate	21.43	1.12
**14**	Panaxydol (fatty alcohols)	13.16	0.71	**36**	Tetraneurin-A-diol	21.75	1.09
**15**	Gibberellic acid<br>Methyl 4,6-tetradecadiynoate	13.26	0.73	**37**	β-sitosterol<br>α-Farnesene	23.36	3.80
**16**	3-Methyl-4-(2,6,6-trimethyl-2-cyclohexen-1-yl)-3-buten-2- one	13.45	1.91	**38**	Oleic Acid<br>9-Octadecenoic acid	23.47	2.53
**17**	3,5-Di-t-butyl-1,4-dihydro- phenacetate	13.64	10.24	**39**	cis-Vaccenic acid<br>Oleic Acid	25.57	1.50
**18**	Cholestan-6-one	15.88	2.78	**40**	Hexadecadienoic acid, methyl ester	27.51	1.28
**19**	3-Ethyl-3-OH-androstan-17-one	16.75	1.82	**41**	2-Methylenecholestan-3-ol	31.05	1.30
**20**	α-N-normethadol	17.02	1.96	**42**	Trielaidin	34.35	1.01
**21**	Dotriacontane	17.33	0.82	**43**	1,25-Dihydroxyvitamin D3	35.53	3.58
**22**	(E)-α-bergamotene	17.83	1.53				

References

22. Elshaarawy, R.F.; Ismail, L.A.; Alfaifi, M.Y.; Rizk, M.A.; Eltamany, E.E.; Janiak, C. Inhibitory activity of biofunctionalized silver-capped N-methylated water-soluble chitosan thiomer for microbial and biofilm infections. *Int. J. Biol. Macromol.* **2020**, *152*, 709–717.
25. Kamal, I.; Khedr, A.I.M.; Alfaifi, M.Y.; Elbehairi, S.E.I.; Elshaarawy, R.F.M.; Saad, A.S. Chemotherapeutic and chemopreventive potentials of ϱ-coumaric acid—Squid chitosan nanogel loaded with Syzygium aromaticum essential oil. *Int. J. Biol. Macromol.* **2021**, *188*, 523–533. https://doi.org/10.1016/j.ijbiomac.2021.08.038.
